# Intellectual Capital and Financial Performance: Comparison With Financial and Pharmaceutical Industries in Vietnam

**DOI:** 10.3389/fpsyg.2021.595615

**Published:** 2021-03-25

**Authors:** Xiao-Bing Zhang, Tran Phuong Duc, Eugene Burgos Mutuc, Fu-Sheng Tsai

**Affiliations:** ^1^Business School, Huaiyin Institute of Technology, Huai'an, China; ^2^CMC Technology & Solutions, Hanoi, Vietnam; ^3^College of Business Administration, Bulacan State University, Malolos, Philippines; ^4^North China University of Water Resources and Electric Power, Zhengzhou, China; ^5^Department of Business Administration, Cheng Shiu University, Kaohsiung, Taiwan; ^6^Center for Environmental Toxin and Emerging-Contaminant Research, Cheng Shiu University, Kaohsiung, Taiwan; ^7^Super Micro Mass Research and Technology Center, Cheng Shiu University, Kaohsiung, Taiwan

**Keywords:** intellectual capital, financial performance, industry comparison, vietnam, pharmaceutical industry

## Abstract

This study investigates the impacts of intellectual capital through Value-Added Intellectual Capital (VAIC) and its components: human capital efficiency (HCE) and structural capital efficiency (SCE) on financial performance in terms of return on assets (ROA) and return on equity (ROE). In addition, this study compares the effects between firms from financial and pharmaceutical industries. A total of 149 Vietnamese firms comprising of 108 financial firms and 41 pharmaceutical firms were examined. Based on the findings, VAIC and HCE show beneficial impacts on both financial performance measures, ROA, and ROE. However, SCE shows adverse and beneficial implications on ROA and ROE, respectively. In terms of industry comparison, VAIC has positive effects on ROA and ROE among the firms from financial industry, whereas it has no effect in the firms from pharmaceutical industry. The effect of HCE on ROA is stronger in the firms from financial industry than firms from pharmaceutical industry while the effect of HCE on ROE is stronger in the firms from pharmaceutical industry than firms from financial industry. The effect of SCE on ROA is stronger in the pharmaceutical firms than financial firms while the effect of SCE on ROE is stronger in the financial firms than pharmaceutical firms. Lastly, the implications of the importance of knowledge-based resources on value creation were elaborated.

## Introduction

Business firms all over the world face the biggest and fiercest competition, nowadays. In order to survive in that dynamic business environment, firms must find various strategies to allocate and develop their resources more efficiently. This action provides a better foundation to enhance market strategies and market performances (Grant, 1991). Valuable resources are often rare, non-substitutable, and inimitable which empower a firm to sustain its competitive advantage and outperform its competitors (Barney, 1991).

The intangible resources are recognized as the most demanding factors for success (Winter and Szulanski, 2002). The physical capital can easily be purchased from the open market (Goh and Ryan, 2002). Compared to the financial-based tangible resources (e.g., manufacturing equipment, real estates, factories, or financial property), the intangible resources of a company (e.g., worker skills, relationship with customers, corporate culture and values, reputation, and organizational structures) are unquestionably more difficult to imitate by its rivals (Sveiby, 1997). Hamel and Prahalad (1990) described these resources as “core competencies” of an organization. The capability to exploit these resources deliberately form the bottom line of intellectual capital (IC) (Bontis, 1998).

IC is present in every business sectors. Companies from different industries are shifting from the traditional production processes to a modernized knowledge business operation, and from blue-collar workers to more knowledgeable human capital (Lipunga, 2014). The most successful ones often possess an IC equal to around from 10 to 20 times their physical assets' value (Roos et al., 2005). Hence, it is certain that IC has become the most important factor in the maintaining firm's competitive advantages and value creation (Shih et al., 2010; Maditinos et al., 2011).

The impacts of IC stands highly challenging despite of its importance (Choo and Bontis, 2002). Scholars in the field have high interest in evaluating IC to provide its implications on financial performance. Furthermore, IC is a very popular topic among the scholars in the developed economies while there are very few studies in the emerging countries (Kamath, 2007). For instance, Cabrilo et al. (2009) mentioned that in Serbia, awareness of IC importance, nature, and its measuring methods was not enough. These concerns raise a gap needing to be filled because the globalization requires all organizations, both from developed and emerging economies, to confront the increasing global competition (Muhammad and Ismail, 2009). This notion makes IC as a relevant factor for the global firms to survive and create an equal demand to promote research in developing countries.

An empirical study was developed to address the gaps in the literature and address this important issue. This study investigates the impact of knowledge-based resources on firm performance, aiming to provide insight among the firms from developing economy. Specifically, this study sought answers on: How does IC relates on financial performance of firms from financial and pharmaceutical industries in Vietnam? A good research of IC about this country may give great suggestions and solutions for other developing countries to follow, in order to achieve stronger economic development. According to World Bank (2014), Vietnamese GDP raised from 77.41 to 193.6 billion USD between 2007 and 2015, which was an upturn of 150.1%. During the same period, the GDP per capita (PPP) of Vietnam increased from 3,907 to 5,668 USD (World Bank, 2014).

Moreover, this study investigates the effects of individual components of IC on financial performance. There were concerns among Vietnamese business community whether the better firms' performance makes citizens richer, or better investment into the human capital of a company staff makes its business better; and whether companies should invest more their employees for great performance. The research uses value-added intellectual capital (VAIC) model which is composed of human capital efficiency (HCE), structural capital efficiency (SCE), and capital employed efficiency (CEE) (Pulic, 2008).

Furthermore, this study compares the impact of IC on financial performance between financial and pharmaceutical industries in Vietnam. Many Vietnamese corporations belong to different industries are doing good business and Vietnamese workers' income are much higher than ever before because of a fast-developing economy. Of all industries in Vietnam, the financial and pharmaceutical ones are receiving governmental focus, due to their importance to the national economy as well as their recent internal problems. Moreover, financial and pharmaceutical are high knowledge intensive industries, which provide a rich environment and reliable financial data for any research.

The outcome of this research provides contribution in the field of knowledge-based resources and value creation. Based on the study findings, management can gain deeper understanding in the relationship between IC, together with its components and firms' financial performance. This research also gives suggestions about the importance of IC to business in general, and financial and pharmaceutical firms in particular, especially in the second-world countries and emerging markets like Vietnam. Furthermore, this research brings information and data for further studies in the future.

## Literature Review and Hypotheses Development

The advent of knowledge-based economy makes the traditional sources of firm's competitive advantage begun to erode (Pablos, 2002). Pablos (2002) mentioned that the existing traditional source depends on tangible assets to sustain competitive advantage and create firm value. Therefore, the essence of IC is the value creation that can be understood as a complex of intangible property, knowledge, skills, processes, applied experience, and technologies used in organizations to ensure a competitive advantage on the market (Papula and Volná, 2011). There are various scholars sharing the same opinion that intellectual capital can help companies increase financial performance. Barney (1991) opined that IC is an important strategic asset with the ability to generate sustainable competitive advantage and superior financial performance. Stewart (1997), Sullivan (2000), and Khanhossini et al. (2013) think of IC as the knowledge that can be used to create wealth, to increase profit and value creation for the company. Other scholars also consider IC as a primary source of value creation in the new economy. Reed et al. (2006) argue that it is only the IC deserving to be considered as strategic resource to create value added for a firm. Although many research confirmed positive relationship between IC and firm performance (Chen et al., 2005; Clarke et al., 2011; Alipour, 2012; Mondal and Ghosh, 2012), some of the results are ambiguous (Javornik et al., 2012; Iazzolino and Laise, 2013).

Most of past research have proved that IC positively affects financial performance (Baroroh, 2013; Poraghajan et al., 2013). Many other research has also proved that the IC has positively implicates on corporate value as measured by the share price both in the developed and developing markets (Ming, 2012; Poraghajan et al., 2013). Many research in the developed countries, such as Singapore, Hongkong, and Taiwan, found positive relationships between IC and financial performance (Chen et al., 2005; Tan et al., 2007). In the emerging countries such as Africa, Iran, Turkey, Greece, China, Indonesia, the phenomenon shows similar findings (Firer and Williams, 2003; Maditinos, 2009; Setayesh and Kazemnejad, 2010; Yi et al., 2011; Pasaribu et al., 2012; Sen, 2014; Tsai and Mutuc, 2020). Recent studies showed similar findings. For instance, Moh'd Khier Al Momani et al. (2020) analyzed the implication of IC and firm performance of the industrial sector in the Amman Stock Exchange (ASE) over the period 2008–2017. Their findings revealed positive relationship between IC and financial performance measures such as market to book value and earnings per share. Their results about the IC components showed mixed findings. They conjectured that the industrial companies in Jordan must reflect on the practical and knowledge experiences because of its essence to strengthen the competitive advantage of the company and lessen the rate of unemployment by hiring expert and skillful employees. There are few research, however, rejecting the positive influences of IC on financial performance (Iswati and Muslich, 2007; Puntillo, 2009; Momani and Nour, 2019). Puntillo (2009) failed to prove the influences of IC on financial performance for the banks listed in Milan stock exchange. Moreover, Momani and Nour (2019) examined the impact of IC on the return of equity ratio (ROE) of commercial banks in Amman Stock Exchange (ASE) during the period 2010–2015. Their findings revealed that IC has negative impact and ROE of commercial banks in Jordan. They mentioned that these banks should be concerned in the IC especially human capital.

The differences in results of how IC affects financial performance leave a room for researcher to carry on doing studies in different regions in the world. Many studies highlighted above related to the developing economies, showing the demand to study IC, and financial performance in the second world economies. That is one of the reason why developing countries like Vietnam is a good location to study IC. Another gap that leaves a room for doing research on IC is the different results among previous studies about the influences of IC components on firms' financial performance.

The VAIC method was also used to explain the phenomenon. Mavridis (2004) mentioned that among the Japanese banks, the strongest performance belonged to those who were most efficient in their application of human capital, whereas physical assets utilization was less important. Yalama and Coskun (2007) conducted a study on the effect of IC profitability of Turkish banks and the result showed that VAIC model can be used as a benchmark for different intellectual efficiency levels. Zehri et al. (2012) investigated the relationship between IC and financial performance in Tunisia. VAIC model was used to measure IC efficiency, while performance of the organization was measure in three ways including financial performance (ROA), market performance (market to book ratio), and economic performance (operating margin). The study later confirmed an effect on corporate financial and economic performance, whereas the relationship between IC and market performance was not confirmed. Ahangar (2011) examined IC and firm performance in Iranian corporate sector. VAIC model was used again to measure IC efficiency and the used performance indexes are profitability, sales growth and employee productivity. Human capital was found out to be the most important component of IC and all of three VAIC dimensions are significant explanatory variables for profitability measured by ROA.

In Southeast Asia, there are many research that have the same confirmation of IC influencing financial performance. Goh (2005) investigated the IC of Malaysian commercial banks using VAIC and found that there was significant relationship between VAIC and HCE. Same findings are revealed by Joshi et al. (2010) when examining the impact of IC on financial performance of Australian Owned Banks for the period from 2005 to 2007. Their studies also show that HCE has relatively larger contribution in measuring VAIC™ performance compared to SCE and CEE. In another study involving different sectors, Muhammad and Ismail (2009) examined the impact of IC efficiency on the performance of Malaysian financial sector firm. By using VAIC to measure IC efficiency and ROA along with profitability to measure performance, the study found a strong positive impact of IC efficiency on the financial performance of Malaysian financial sector. Moreover, Malaysian banking sector relies more heavily on the IC efficiency, followed by insurance sector and brokerage firms subsequently. Furthermore, in Malaysia, Ting and Lean (2009) conducted the study on financial sector to examine the relationship between IC performance and financial performance for the period from 1999 to 2007. VAIC is also used, and the results insisted that IC and ROA are positively related and the three components of VAIC were able to explain 71.6 per cent of profitability.

Phusavat et al. (2011) targeted Thai manufacturing firms to conduct a research on the effects of IC, and integrated its key components and performance using VAIC. The study revealed that IC positively and significantly affected manufacturing firms' performance, having influences on the all four performance indicators under examination, i.e., ROE, ROA, revenue growth, and employee productivity. The finding of these studies yield mixed results. For example, the results of the study about South African companies by Firer and Williams (2003) only supported IC and capital employed. Furthermore, the relationship between IC and traditional measures of firm performance (ROA, ROE), are failed to be confirmed. The opposite research result, studied by Iswati and Muslich (2007), showed no relationship between IC and financial performance in Jakarta Stock Exchange.

Aware of the above-mentioned gaps in the literature, it is clear that almost all studies are related to firms in a specific country context, and there are very few studies that included inter-industry comparisons. IC and firms' financial performance seem to be related to some extent in many studies, but the results were not consistent; therefore, the results cannot be generalized. In Vietnam, an important emerging market and developing country of the world, the study about the impacts of IC on firms' financial performance, both in English and in Vietnamese language, are very scarce. This leaves another gap to do a study in Vietnam. Hence, the following hypotheses are developed to examine the relationship between IC and firms' financial performance, using data from Vietnamese financial and pharmaceutical firms. The sub-hypotheses H_1a_ and H_1b_ are derived from the fact that this research will use VAIC and its components to measure IC.

H1: IC has significantly positive impacts on Vietnamese firms' financial performance.
H_1a_: HCE has significantly positive impacts on Vietnamese firms' financial performance.
H_1b_: SCE has significantly positive impacts on Vietnamese firms' financial performance.

### Intellectual Capital in Financial and Pharmaceutical Firms

The Vietnamese financial system is too large for a low middle-income country, with assets of more than 200 percent of GDP in 2011. The primary players in Vietnamese financial industries are banks and financial institutions. While the financial system is too large compared to other countries, its expansion has been volatile in recent years, reflecting the unstable external environment and erratic macroeconomic policies.

Due to 2008 global economic crisis, credit, and economic activity slowed down at the end of that year, leading to the need of having a corrective response from the governments. Furthermore, due to the easy fiscal and monetary policies, another credit spike happened in 2009 and 2010, the policy then was tightened, and a bad credit slowdown appeared in 2011. Banking specialists raised their voices about the problem of excessive tightening led to another aggressive loosening of policies in 2012, in which policy rates and many other administrative measures were cut. Despite the try of policy easing, credit growth stayed stagnant. Although the macroeconomy has stabilized since 2012, financial vulnerabilities still need a better solution. Recently, Vietnamese economy has different symptoms of corporate and financial disappointment, and the growth appeared to be weaker. Several corporate segments displayed poor financial performance, and have badly hurtled the financial system. Large state-owned enterprises (SOEs) have defaulted on their obligations and several others appear to be overleveraged. At the end of 2012, Vietnamese banking system has accumulated many bad debts (around 12 percent of total loans), therefore, many small banks have had to face more serious liquidity and solvency problems, leading to interventions by the State Bank of Vietnam (SBV).

In terms of pharmaceutical industries, drugs and medicines are products with direct influence on human health, which makes their demand inelastic to the price. With the growing conception of Vietnamese people on healthcare and medical need, the profit in medicine industry has been keeping increasing dramatically. The average revenue growth from 2009 to 2013 reached 18.8% per annum. This growth was regardless of other economic sectors during the economic crisis from 2008 to 2013 (VietinbankSc Industry Report, 2014). Due to the better quality of Western medicine, the pharmaceutical import in Vietnam worth USD 2.15 billion in 2013, while export businesses worth only USD 100 million. That leads to the government attention to think about investing more into the research and development departments of Vietnamese pharmaceutical firms.

All over the world, the gaps between the book value and market value are considered more significant in knowledge-based firms such as financial and pharmaceutical firms, due to their large proportion of intangible assets, intellectual property, and IC (Edvinsson and Malone, 1997; Shih et al., 2010). Therefore, many studies about intellectual capital in such kinds of firms were conducted. In the pharmaceutical industry, the studies in Germany, Jordan, and Taiwan provided empirical evidences about impacts of human capital and structural capital on firms' performance (Bollen et al., 2005; Huang and Wu, 2010; Sharabati et al., 2010). In financial industry, many studies also proved the similar effects of intellectual capital (Reed et al., 2006; Samiloglu, 2006). There are also arguments that an efficient utilization of intellectual capital is more crucial for accomplishing success in finance and banking than in other industries, asserting that delivering of high-quality services by a bank depends on its investment in items related to IC such as its human resources, brand building, systems, and processes (Ahuja and Ahuja, 2012). But yet, there is lack of empirical evidences to prove the point, which leaves room for scholars to deepen. Because of the importance of the two industries in Vietnamese national economy, the lack of studies of the field in the country, and the literature gap, the second main hypothesis are proposed to be tested in order to compare the impacts of intellectual capital on financial performance between Vietnamese financial and pharmaceutical firms.

H2: IC has stronger impacts on financial performance of Vietnamese financial firms than Vietnamese pharmaceutical firms.
H2a: HCE has stronger impacts on financial performance of Vietnamese financial firms than Vietnamese pharmaceutical firms.
H2b: SCE has stronger impacts on financial performance of Vietnamese financial firms than Vietnamese pharmaceutical firms.

## Methodology

This study examines the impact of IC and its components (human capital and structural capital) on financial performance of financial firms (banks and financial institutions) and pharmaceutical firms. Only firms which are active for more than 5 years were considered in this study. Young entrepreneurial companies were excluded because their values is better described in the market value (i.e., stock price, earning per share) rather than financial performance.

We obtained a final sample of 149 companies. It is composed of 108 financial firms and 41 pharmaceutical firms. These companies were actively listed accordingly in the National Portal of Business Registration. The financial data for the year 2016 were collected to analyze the phenomenon. These data were matched with complete information for the computation of IC estimate.

### Measure of VAIC

Pulic (1998, 2000a,b) developed VAIC™ as a measurement system based on the real value and performance of a corporation, region, or nation for helping in benchmarking and predicting future capabilities. There are five steps to calculate intellectual capital using VAIC model (Muhammad and Ismail, 2009).

Step 1: Calculation of Value Added (VA)

VA = OUT – IN

where OUT is the total income from all products and services sold, IN is the expenses (except labor, taxation, interest, dividends, depreciation) of firm.

Step 2: Calculation of CEE

CEE = VA / CE

where CEE is the value created by one unit of capital employed, and CE is the Capital Employed measure by the total tangible assets.

Step 3: Calculation of HCE

HCE = VA / HC

where HCE is the value created by one unit of Human Capital invested, HC is Human Capital measured based on the total salaries, wages, and all incentives of employees.

Step 4: Calculation of SCE

SCE = SC / VA

where SCE is the proportion of total VA accounted by structural capital, SC is the Structural Capital calculated based on the difference between VA and HC.

Step 5: Calculation of VAIC

VAIC = CEE + HCE + SCE

VAIC indicates corporate value creation efficiency on firm resources, whereas the sum of HCE and SCE is equal to Intellectual Capital Efficiency (ICE).

### Financial Performance Measures

Financial performance is the level of business or firm performance over a specific period of time, expressed in terms of overall profits and deficits during that time. Evaluating financial performance of a company helps decision-makers determine the efficiency of different levels of business strategies. This research reflects on some ratios in the technique of ratio analysis, because other techniques of financial analysis are not appropriate in the context. Among many profitability ratios, ROA and ROE are the two considered by many experts as most important ones showing exact a firms' financial performance. ROA shows the ability of a firm to generate profits from its assets, while ROE reveals how much profit a firm can generate with the physical investment. In the economic context of an emerging market like Vietnam, where the companies often have big investment funds in a short period of time, ROA and ROE proved to be the most suitable ones to be examined. ROA and ROE were used as the indicators to give a clear picture of how IC affects firm financial performance. Many other studies of the field, which will be mentioned later in this research, used the same indicators.

### Regression Models

We employ multivariate regression to examine the association of IC and its components on financial performance. A control variable about the size of the company will be added in to test about the differences between measures of dependent and independent variables. We use the natural logarithm of total assets as proxy of size. According to Article 3 of Decree Number 56/2009/ND-CP (June 30th, 2009) of Vietnamese Government, the small and medium companies have total asset of under 100 billion Vietnam Dong, while the large ones have total asset of more than 100 billion Vietnam Dong. Moreover, this study controls for industry and considers dummy variable IND_Dummy, which represents 1 if the firms is from financial industry, and 0 if from pharmaceutical industry. The following models were estimated:

(1) $$\begin{array}{r} FP_{i,t}\;=\;\alpha \;+\;\beta_{1}\;VAIC_{i,t}\;+\;\beta_{2}\;SIZE_{i,t}\\ \;+\;\beta_{3}\;{Ind\text{\_}Dummy}_{i,t}+\varepsilon_{i,t} \end{array}$$

(2) $$\begin{array}{r} FP_{i,t}\;=\;{\alpha \;}_{1}\;+\;\beta_{1}^{a}\;HCE_{i,t}\;+\;\beta_{2}^{a}\;SIZE_{i,t}\\ \;+\;\beta_{3}^{a}\;{Ind\text{\_}Dummy}_{i,t}\;+\varepsilon_{i,t} \end{array}$$

(3) $$\begin{array}{r} FP_{i,t}\;=\;{\alpha \;}_{2}\;+\;\beta_{1}^{b}\;SCE_{i,t}\;+\;\beta_{2}^{b}\;SIZE_{i,t}\\ \;+\;\beta_{3}^{b}\;{Ind\text{\_}Dummy}_{i,t}\;+\varepsilon_{i,t} \end{array}$$

## Results and Discussions

Table 1 reports the descriptive values of each variable. Table 1 shows that the VAIC of the combined samples have 4.15 mean value, whereas firms from pharmaceutical industry show higher mean value of 5.98 than firms from financial industry with 3.46. Similarly, pharmaceutical firms have greater HCE and SCE than financial firms. From the values of means, we can see that among three components of VAIC, HCE is the main one. Human capital is the major focus of Vietnamese financial and pharmaceutical firms, while the contribution of Structural Capital is small. The combined samples show an average values of 0.02 and 0.06 in terms of ROA and ROE. Pharmaceutical firms show greater financial performance that financial firms. Moreover, Table 1 shows that the mean difference between financial and pharmaceutical firms are statistically significant at *p* < 0.01 for VAIC, HCE, and SIZE.

**Table 1:** Descriptive statistics.

	**Overall**	**Financial**	**Pharmaceutical**	***p* value**
ROA	0.02	0.01	0.06	0.17
	(0.18)	(0.18)	(0.18)	
ROE	0.06	0.04	0.12	0.22
	(0.34)	(0.36)	(0.28)	
VAIC	4.15	3.46	5.98	0.00^***^
	(4.56)	(4.77)	(3.35)	
HCE	3.00	2.50	4.31	0.00^***^
	(3.53)	(3.59)	(3.03)	
SCE	0.86	0.85	0.90	0.91
	(2.79)	(3.15)	(1.49)	
SIZE	13.93	14.26	13.06	0.00^***^
	(2.04)	(2.19)	(1.22)	
IND_Dummy	0.72		1.20	
	(0.45)			
N	149	108	41	

*Values per column are the mean values while standard deviation values are in parenthesis*.

*** *Is statistical significance at the 1% levels on a two-tailed test*.

Table 2 presents the results of correlation analysis. ROA shows high correlation on ROE. This study consider two measures of financial performance. Hence, a separate regression analyses were conducted. Both financial measures show weak correlation to the independent variables of the study. Moreover, as expected, VAIC has high correlation on its components HCE and SCE. To avoid multicollinearity issue, this study run a separate regression to each component.

**Table 2:** Correlation among variables.

	**ROA**	**ROE**	**VAIC**	**HCE**	**SCE**	**SIZE**	**IND_Dummy**
ROA	1.00						
ROE	0.85^***^	1.00					
VAIC	0.21^**^	0.39^***^	1.00				
HCE	0.41^***^	0.34^***^	0.79^***^	1.00			
SCE	−0.25^***^	0.14	0.56^***^	−0.06	1.00		
SIZE	0.10	0.12	−0.17^**^	−0.11	−0.07	1.00	
IND_Dummy	−0.11	−0.10	−0.25^***^	−0.23^***^	−0.01	0.26^***^	1.00

*** *Correlation is significant at the 0.01 level (2-tailed)*.

** *Correlation is significant at the 0.05 level (2-tailed)*.

Table 3 shows the findings of the multiple regressions of ROA on VAIC and its components in the whole sample and sub-samples from financial and pharmaceutical industries. Results from combined sample show that VAIC is positively and significantly associated to ROA at (*p* < 0.05), indicating that firms with high-VAIC investments generate better financial performance. This finding supports the hypothesis H1. HCE shows similar findings at (*p* < 0.01), indicating that firms with high-HCE investments generate better financial performance. This finding supports the hypothesis H1a. The findings in SCE, however, show negative effect on ROA at (*p* < 0.01), indicating that firms with high-SCE investments generate lower financial performance. This finding rejects the hypothesis H1b.

**Table 3:** Multiple regressions of ROA on VAIC and its components.

	**Overall**			**Financial**			**Pharmaceutical**		
VAIC	0.21^**^			0.31^***^			−0.17		
	(2.48)			(3.34)			(−0.85)		
HCE		0.42^***^			0.47^***^			0.44^**^	
		(5.44)			(5.48)			(2.41)	
SCE			−0.24^***^			−0.13			−0.92^***^
			(−3.02)			(−1.35)			(−14.03)
SIZE	0.16^*^	0.16^**^	0.12	0.23^**^	0.24^***^	0.15	0.11	−0.24	0.12^*^
	(1.93)	(2.05)	(1.46)	(2.47)	(2.78)	(1.60)	(0.56)	(−1.32)	(1.86)
IND_Dummy	−0.10	−0.06	−0.15^*^						
	(−1.22)	(−0.74)	(−1.78)						
F	3.64^**^	11.70^***^	4.66^***^	7.21^***^	16.93^***^	2.42^*^	0.36	2.91^*^	98.36^***^
Adj. *R*^2^	0.05	0.18	0.07	0.10	0.23	0.03	−0.03	0.09	0.83

*Values per column are the mean values while standard deviation values are in parenthesis*.

*** *Is statistical significance at the 1% levels on a two-tailed test*.

** *Is statistical significance at the 5% levels on a two-tailed test*.

* *Is statistical significance at the 10% levels on a two-tailed test*.

Table 3 shows that VAIC has a positive and significant effect on ROA at (*p* < 0.05) among the firms from financial industry, whereas it has no effect in the firms from pharmaceutical industry. This finding supports H2 which indicates that the effect of IC is stronger in the financial firms than pharmaceutical firms. In addition, Table 3 shows that HCE has positive and significant effect on ROA of both industries. Hence, our hypothesis testing from H2a requires additional testing to check if the effect of HCE is greater in the financial firms than pharmaceutical firms. The SCE has negative and significant effect on ROA at (*p* < 0.01) in the firms from pharmaceutical industry, whereas it shows no effect in the firms from financial industry. This finding rejects H2b which states that the effect of SCE is stronger in the financial firms than the pharmaceutical firms.

Table 4 shows the findings of the multiple regressions of ROE on VAIC and its components in the whole sample and sub-samples from financial and pharmaceutical industries. Results from combined sample show that VAIC is positively and significantly associated to ROE at (*p* < 0.01), indicating that firms with high-VAIC investments generate better financial performance. This finding supports the hypothesis H1. HCE and SCE show similar findings at (*p* < 0.01) and (*p* < 0.10), indicating that firms with high-HCE and SCE investments generate better financial performance. This finding supports the hypothesis H1a and H1b.

**Table 4:** Multiple regressions of ROE on VAIC and its components.

	**Overall**			**Financial**			**Pharmaceutical**		
VAIC	0.41^***^			0.54^***^			−0.16		
	(5.34)			(6.46)			(−0.79)		
HCE		0.35^***^			0.38^***^			0.38^**^	
		(4.39)			(4.26)			(2.06)	
SCE			0.15^*^			0.30^***^			−0.91^***^
			(1.90)			(3.29)			(−12.68)
SIZE	0.21^***^	0.18^**^	0.17^**^	0.29^***^	0.24^***^	0.21^**^	0.12	−0.20	0.13^*^
	(2.67)	(2.26)	(2.08)	(3.54)	(2.68)	(2.27)	(0.59)	(−1.05)	(1.85)
IND_Dummy	−0.05	−0.07	−0.15^*^						
	(−0.68)	(−0.85)	(-1.75)						
F	11.59^***^	8.41^***^	3.00^**^	23.36^***^	11.14^***^	7.35^***^	0.32	2.13	80.40^***^
Adj. *R*^2^	0.18	0.13	0.04	0.29	0.16	0.11	−0.04	0.05	0.80

*Values per column are the mean values while standard deviation values are in parenthesis*.

*** *Is statistical significance at the 1% levels on a two-tailed test*.

** *Is statistical significance at the 5% levels on a two-tailed test*.

* *Is statistical significance at the 10% levels on a two-tailed test*.

Table 4 shows that VAIC has a positive and significant effect on ROE at (*p* < 0.01) among the firms from financial industry, whereas it has no effect in the firms from pharmaceutical industry. This finding supports H2 which indicates that the effect of IC is stronger in the financial firms than pharmaceutical firms. In addition, Table 3 shows that HCE has positive and significant effect on ROE of both industries. Hence, our hypothesis testing from H2a requires additional testing to check if the effect of HCE is greater in the financial firms than pharmaceutical firms. The SCE has positive and significant effect on ROE at (*p* < 0.01) in the firms from financial industry, whereas it shows negative and significant effect on ROE at (*p* < 0.01) in the firms from pharmaceutical industry. This finding supports H2b which states that the effect of SCE is stronger in the financial firms than the pharmaceutical firms.

Result from this additional testing for H2b is presented in Table 5. We use the following t-statistics to calculate the difference between any two estimated coefficients (Lee et al., 2013). We calculate t-statistics based on the following equation: *t* = (βA - βB) / $\sqrt{\sigma_{A}^{2}/\;n_{A}-\sigma_{B}^{2}/\;n_{B}}$ where A is the financial firms, B is the pharmaceutical firms β is the beta coefficient, σ^2^ is the SE of an estimated coefficient and n is the number of observations.

**Table 5:** Summary of hypotheses testing results for financial and pharmaceutical industries.

**Hypothesis testing**	**Description**	***t*-statistics**	**Results**
H_2b_ for ROA	The effect of HCE on ROA is stronger in the firms from financial industry than firms from pharmaceutical industry.	19.45	Supported
H_2b_ for ROE	The effect of HCE on ROE is stronger in the firms from financial industry than firms from pharmaceutical industry.	−6.27	Not supported

*Summary result of t-tests to examine if the coefficients are statistically significant different along high-CSR ratings group and low-CSR ratings group*.

Table 5 shows that regarding ROA, estimated coefficients under financial and pharmaceutical firms are 0.47 and 0.44, respectively. These findings suggest that ROA of firms in the financial industry is 47% for every HCE value while 44% to firms in the pharmaceutical industry. The corresponding t-statistic is 19.45 suggesting that the coefficient under firms in the financial industry is higher than firms in the pharmaceutical industry. Hence, our result reveals that the effect of HCE on ROA is stronger in the firms from financial industry than firms from pharmaceutical industry, consistent with H2b.

Table 5 shows that regarding ROE, estimated coefficients under financial and pharmaceutical firms are 0.38 and 0.38, respectively. These findings suggest that ROE of firms in the financial industry is 38% for every HCE value while 38% to firms in the pharmaceutical industry. The corresponding t-statistic is −6.27 suggesting that the coefficient under firms in the financial industry is lower than firms in the pharmaceutical industry. Hence, our result reveals that the effect of HCE on ROE is stronger in the firms from pharmaceutical industry than firms from financial industry, inconsistent with H2b.

## Conclusions

This study investigates the impact of IC on financial performance of Vietnamese firms. In addition, this study compares the effects of IC and its components on financial performance between firms from financial and pharmaceutical industries. Based on the findings, VAIC and HCE show beneficial impacts on both financial performance measures, ROA and ROE. However, SCE shows adverse and beneficial implications on ROA and ROE, respectively. In terms of industry comparison, VAIC has positive effects on ROA and ROE among the firms from financial industry, whereas it has no effect in the firms from pharmaceutical industry. The effect of HCE on ROA is stronger in the firms from financial industry than firms from pharmaceutical industry while the effect of HCE on ROE is stronger in the firms from pharmaceutical industry than firms from financial industry. The effect of SCE on ROA is stronger in the pharmaceutical firms than financial firms while the effect of SCE on ROE is stronger in the financial firms than pharmaceutical firms.

This study provides several contributions in the field of intellectual capital and financial performance. Theoretically, the outcome of this research contributes in the field of knowledge-based resources and value creation through the investigation of the implications of IC and its components on financial performance and the industry comparison of the phenomenon. Based on the study findings, management can gain deeper understanding in the relationship between IC, together with its components and firms' financial performance. This research also gives suggestions about the importance of IC to business in general, and financial and pharmaceutical firms in particular, especially in the second-world countries and emerging markets like Vietnam.

Belonging to a knowledge-intensive and skill-based industry, banks, insurance companies, or investment funds are ideal organizations to study and implement intellectual capital. In Vietnam, however, there is no major study about intellectual capital yet, in any industry. Vietnamese financial firms' managers in general also do not possess much expertise in this field, which wastes corporate potential to build stronger competitive advantages. This study has proved a linear relationship between IC and financial performance, but Vietnamese firms will surely question about the real application of a new term in their organization. Since Vietnamese financial firms often copy the business models and methods from Western countries to do their business, regardless of differences in business and culture environments, appropriate investment into intellectual capital will benefit both market performance and financial performance of enterprises. More human capital efficiency will enable financial firms for more innovation in investment derivative models, or in risk management, which can help achieving more profit and brand image. More structural capital efficiency will give firms more balance between formal control and informal control, which can foster human capital efficiency and firms' performance. In addition, managers can use IC as a tool to evaluate the performance of their corporations, enabling them to understand the importance of training and educating creative employees (Cabrita and Bontis, 2008).

Similar to financial industry, pharmaceutical is knowledge-intensive and skill-based. There are studies in Germany, Jordan, and Taiwan provided empirical evidences about impacts of human capital and structural capital on the performance of pharmaceutical firms (Bollen et al., 2005; Huang and Wu, 2010; Sharabati et al., 2010), but again, in Vietnam, there is no major study. In Vietnam, due to the fact that the medicines produced by domestic firms are considered not as good as imported medicines, investment on patented medicines is necessary. A beneficial relationship between human capital and financial performance in Vietnamese pharmaceutical industry is confirmed by this study, which gives companies more evidences to invest into innovation, research, and development. When a pharmaceutical firm is too structural in their organization, the flexibility its people need for invention and innovation may disappear, lead to the constraint in developing human capital. Therefore, Vietnamese pharmaceutical firms should work out to reach the peak of the structural capital curve and stay there, where the most structural capital efficiency locates.

This research has several pitfalls. First, the information about intellectual capital through VAIC model employed in this study are not free of measurement issues. These limitations include conceptual vagueness, the ability of the estimate to present the potential value creation, and the ability of the model to incorporate the IC components interactions (Ståhle et al., 2011; Janoševi et al., 2013). This measurement considers financial information relevant to human capital and structural capital. VAIC model has been utilized by researchers in the field who have been trying to analyze the phenomenon about IC and financial performance despite of the its drawbacks (Zeghal and Maaloul, 2010; Janoševi et al., 2013). Second, similar to the past literature, the sociodemographic data were not described and assess in this study, which may be the subject of future research. Third, this study only reflected on the previously published articles and excluded unpublished literature explaining the phenomenon. The supporting concepts and foundations of the study from the published articles were used as motivation of the present study. Moreover, this study employs quantitative research design to explore the link between IC and financial performance. A mixed-method study is recommended to analyze the qualitative components of the relationship to explain the inconclusive findings in the past. Furthermore, future studies may investigate IC and financial performance across different industries and make comparative analysis across industries in Vietnam or in a cross-border analysis. The investment of firms on IC may vary across different countries and industries, based on practice, orientation, and business operations.

## Data Availability Statement

The raw data supporting the conclusions of this article will be made available by the authors, without undue reservation.

## Author Contributions

F-ST: conceptualization. EB: methodology. F-ST: validation. TD: formal analysis and data curation. X-BZ and TD: writing—original draft preparation. F-ST and EB: writing-review and editing. X-BZ: funding acquisition. All authors have read and agreed to the published version of the manuscript.

## Conflict of Interest

TD was employed by company CMC Technology & Solutions. The remaining authors declare that the research was conducted in the absence of any commercial or financial relationships that could be construed as a potential conflict of interest.

## References

[B1] Ahangar R. G. (2011). The relationship between intellectual capital and financial performance: an empirical investigation in an Iranian company. African J. Business Manage. 5, 88–95. 10.5897/AJBM10.712

[B2] Ahuja B. R. Ahuja N. L. (2012). Intellectual capital approach to performance evaluation: a case study of the banking sector in India. Int. Res. J. Finance Econom. 93, 110–122.

[B3] Alipour M. (2012). The effect of intellectual capital on firm performance: an investigation of Iran insurance companies. Measur Business Excellence 16, 53–66. 10.1108/13683041211204671

[B4] Barney J. B. (1991). Firm resources and sustained competitive advantage. J. Manage. 17, 99–120. 10.1177/014920639101700108

[B5] Baroroh N. (2013). Analysis on the influence of intellectual capital on financial performance manufacturing. Jurnal Dinamika Akuntansi 5, 172–182.

[B6] Bollen L. Vergauwen P. Schnieders S. (2005). Linking intellectual capital and intellectual property to company performance. Manage. Decision 43, 1161–1185. 10.1108/00251740510626254

[B7] Bontis N. (1998). Intellectual capital: An exploratory study that develops measures and models. Manage. Decision 36, 63–76. 10.1108/00251749810204142

[B8] Cabrilo S. Uzelac Z. Cosic I. (2009). Researching indicators of organizational intellectual capital in Serbia. J. Intellect. Capital 10, 573–587. 10.1108/14691930910996652

[B9] Cabrita M. Bontis N. (2008). Intellectual capital and business performance in the Portuguese banking industry. Int. J. Tech Manage. 43, 1–26. 10.1504/IJTM.2008.019416

[B10] Chen M. Cheng S. Hwang Y. (2005). An empirical investigation of the relationship between intellectual capital and firms' market value and financial performance. J. Intellect. Capital 6, 159–176. 10.1108/14691930510592771

[B11] Choo C. W. Bontis N. (2002). The Strategic Management of Intellectual Capital and Organizational Knowledge. New York, NY: Oxford University Press.

[B12] Clarke M. Seng D. Whiting R. (2011). Intellectual capital and firm performance in Australia. J. Intellect. Capital 12, 505–530. 10.1108/14691931111181706

[B13] Edvinsson L. Malone M. S. (1997). Intellectual Capital: Realizing Your Company's True Value by Finding Its Hidden Brainpower. New York, NY: Harper Business.

[B14] Firer S. Williams M. (2003). Intellectual capital and traditional measures of corporate performance. J. Intellect. Capital 4, 348–360. 10.1108/14691930310487806

[B15] Goh P. C. (2005). Intellectual capital performance of commercial banks in Malaysia. J. Intellect. Capital 6, 385–396. 10.1108/14691930510611120

[B16] Goh S. C. Ryan P. J. (2002). “Learning capability, organizational factors and firm performance,” in 3rd European Conference on Organizational Knowledge, Learning and Capabilities (Athens).

[B17] Grant R. M. (1991). The resource-based theory of competitive advantage: implications for strategy formulation. California Manage. Rev. 33, 114–135. 10.2307/41166664

[B18] Hamel G. Prahalad C. K. (1990). The core competence of the corporation. Harvard Business Rev. 68, 79–91.

[B19] Huang Y. C. Wu Y. C. J. (2010). Intellectual capital and knowledge productivity: the Taiwan biotech industry. Manage. Decision 48, 580–599. 10.1108/00251741011041364

[B20] Iazzolino G. Laise D. (2013). Value added intellectual coefficient (VAIC). A methodological and critical review. J. Intellect. Capital 14, 547–563. 10.1108/JIC-12-2012-0107

[B21] Iswati S. Muslich A. (2007). “The influence of intellectual capital to financial performance at insurance Companies in Jakarta Stock Exchange (JSE),” in Proceedings of the 13th Asia Pacific Management Conference (Melbourne, VIC), 1393–1399.

[B22] Janoševi,ć S. DŽenopoljac V. Bontis N. (2013). Intellectual capital and financial performance in Serbia. Knowledge Process Manage. 20, 1–11. 10.1002/kpm.1404

[B23] Javornik S. Tekavcic M. Marc M. (2012). The efficiency of intellectual capital investments as a potential leading indicator. Int. Business Econom. Res. J. 11, 535–558. 10.19030/iber.v11i5.6972

[B24] Joshi M. Cahill D. Sidhu J. (2010). Intellectual capital performance in the banking sector: an assessment of Australian owned banks. J. Human Resource Costing Account. 14, 151–170. 10.1108/14013381011062649

[B25] Kamath G. B. (2007). The intellectual capital performance of Indian banking sector. J. Intellect. Capital 8, 96–123. 10.1108/14691930710715088

[B26] Khanhossini D. Nikoonesbati M. Kheire H. Moazez E. (2013). “Investigating of relationship between intellectual capital and financial performance in MAPNA group companies,” in Working Paper From Azad University, 1–14. 10.2139/ssrn.2216638

[B27] Lee J. S. Yen P. H. Chan K. C. (2013). Market states and disposition effect: Evidence from Taiwan mutual fund investors. Appl. Econom. 45, 1331–1342. 10.1080/00036846.2011.617696

[B28] Lipunga A. M. (2014). A longitudinal assessment of intellectual capital of companies listed on malawi stock exchange. Eur J Business Manage. 6(27–35.

[B29] Maditinos D. (2009). “Intellectual capital and business performance: an empirical study for the Greek listed companies,” in 7th International Conference on Accounting and Finance in Transition (London), 23–25.

[B30] Maditinos D. Chatzoudes D. Tsairidis C. Theriou G. (2011). The impact of intellectual capital on firms' market value and financial performance. J. Intellect. Capital 12, 132–151. 10.1108/14691931111097944

[B31] Mavridis D. G. (2004). The intellectual capital performance of the Japanese banking sector. J. Intellect. Capital 5, 92–115. 10.1108/14691930410512941

[B32] Ming C. C. (2012). An Empirical investigation of the relationship between Intellectual capital and Firm's market value and Financial Performance. Research working paper, Department of Accounting, National Chengchi University, Taipei, Taiwan.

[B33] Moh'd Khier Al Momani K. Jamaludin N. Zanani Wan Abdullah W. Z. Ibrahim Nour A.-N. (2020). The effects of intellectual capital on firm performance of industrial sector in Jordan. Humanit. Soc. Sci. Rev. 8, 184–192. 10.18510/hssr.2020.8222

[B34] Momani K. M. D. Nour A. N. I. (2019). The influence of intellectual capital on the return of equity among banks listed in Amman stock exchange. Int. J. Electronic Banking 1, 220–232. 10.1504/IJEBANK.2019.099613

[B35] Mondal A. Ghosh S. K. (2012). Intellectual capital and financial performance of Indian banks. J. Intellect. Capital 13, 515–530. 10.1108/14691931211276115

[B36] Muhammad N. M. N. Ismail M. K. A. (2009). Intellectual capital efficiency and firm's performance: study on malaysian financial sectors. Int. J. Econom. Finance 1, 206–212. 10.5539/ijef.v1n2p206

[B37] Pablos P. O. (2002). Evidence of intellectual capital measurement from Asia, Europe and Middle East. J. Intellect. Capital 3, 287–302. 10.1108/14691930210435624

[B38] Papula J. Volná J. (2011). “Intellectual capital as value adding element in knowledge management. Knowledge as Business Opportunity,” in Proceeding of the Conference of the International School for Social and Business Studies (Celje, Slovenia).

[B39] Pasaribu H. Dian I. P. Indri T. H. (2012). The role of corporate intellectual capital. Am. Int. J. Contemporary Res. 2, 162–170.

[B40] Phusavat K. Comepa N. Sitko-Lutek A. Ooi K. (2011). Interrelationships between intellectual capital and performance: empirical examination. Industrial Manage. Data Syst. 111, 810–829. 10.1108/02635571111144928

[B41] Pouraghajan A. Ramezani A. Mohammadzadeh S. (2013). Impact of intellectual capital on market value and firms' financial performance: evidences from teheran stock exchange. World Sci. J. 1, 197–208.

[B42] Pulic A. (1998). Measuring the performance of intellectual potential in knowledge economy. Paper presented at the 2nd McMaster World Congress on Measuring and Managing Intellectual Capital by the Austrian Team for Intellectual Potential. Hamilton.

[B43] Pulic A. (2000a). VAIC – an accounting tool for IC management. Int. J. Tech. Manage. 20, 702–714. 10.1504/IJTM.2000.002891

[B44] Pulic A. (2000b). MVA and VAIC Analysis of Randomly Selected Companies From FTSE 250. Available online at: http://www.vaic-on.net

[B45] Pulic A. (2008). The Principles of Intellectual Capital Efficiency - A Brief Description, Croatian Intellectual Capital Center, Zagreb.

[B46] Puntillo P. (2009). Intellectual capital and business performance: evidence from Italian banking industry. Electron J. Corporate Finance 4, 96–115.

[B47] Reed K. K. Lubatkin M. Srinivasan N. (2006). Proposing and testing an intellectual capital-based view of the firm. J. Manage, Stud. 43, 867–893. 10.1111/j.1467-6486.2006.00614.x

[B48] Roos G. Pike S. Fernström L. (2005). Managing Intellectual Capital in Practice. Oxford: Butterworth-Heinemann.

[B49] Samiloglu F. (2006). The performance analysis of the Turkish banks through VAIC and MV/MB ratio. J. Administrat. Sci. 4, 237–257.

[B50] Sen K.I. (2014). Uluslararasi Finansal Raporlama Standartlarina Geçişin Entelektüel Sermaye Üzerindeki Etkileri: Toprak Ve Topraga Dayali Ürünler Sektöründe Bir Araştirma. Çankiri Karatekin Üniversitesi Sosyal Bilimler Enstitüsü Dergisi 5, 89–108.

[B51] Setayesh M. H. Kazemnejad M. (2010). A study on the effect ownership structure and composition of board of directors on dividend policy of listed companies in Tehran stock exchange. J. Account. Knowledge 1, 29–51. 10.22103/jak.2012.54

[B52] Sharabati A. A. A. Jawad S. N. Bontis N. (2010). Intellectual capital and business performance in the pharmaceutical sector of Jordan. Manage Decision 48, 105–131. 10.1108/00251741011014481

[B53] Shih K. Chang C. Lin B. (2010). Assessing knowledge creation and intellectual capital in banking industry. J. Intellect. Capital 11, 74–89. 10.1108/14691931011013343

[B54] Ståhle P. Ståhle S. Aho S. (2011). Value added intellectual coefficient (VAIC): a critical analysis. J. Intellect. Capital 12, 531–551. 10.1108/14691931111181715

[B55] Stewart T. A. (1997). Intellectual Capital: The New Wealth of Organizations. New York, NY: Doubleday/Currency.

[B56] Sullivan P. H. (2000). Value-driven Intellectual Capital: How to Convert Intangible Corporate Assets into Market Value. Toronto: John Wiley and Sons.

[B57] Sveiby K. (1997). The New Organisational Wealth: Managing and Measuring Knowledge Based Assets. San Francisco, CA: Berrett-Kohler.

[B58] Tan H. P. Plowman D. Hancock P. (2007). Intellectual capital and financial returns of companies. J. Intellect. Capital 8, 76–95. 10.1108/14691930710715079

[B59] Ting I. W. K. Lean H. H. (2009). Intellectual capital performance of financial institutions in Malaysia. J. Intellect. Capital 10, 588–599. 10.1108/14691930910996661

[B60] Tsai C. H. Mutuc E. B. (2020). Evidence in Asian food industry: intellectual capital, corporate financial performance, and corporate social responsibility. Int. J. Environ. Res. Public Health 17:663. 10.3390/ijerph17020663 31968553 PMC7014449

[B61] VietinbankSc Industry Report (2014). Vietnam Pharmaceutical Industry.

[B62] Winter S. Szulanski G. (2002). “Replication of organizational routines: conceptualizing the exploitation of knowledge assets,” in The Strategic Management of Intellectual Capital in Organizational Knowledge (eds) C. Choo and N. Bontis (New York, NY: Oxford University Press), 207–21.

[B63] World Bank (2014). Vietnam - Financial Sector Assessment. Financial Sector Assessment Program (FSAP). Washington, DC, World Bank Group. Available online at: http://documents.worldbank.org/curated/en/216401468329363389/Vietnam-Financial-sector-assessment

[B64] Yalama A. Coskun M. (2007). Intellectual capital performance of quoted banks on the Istanbul stock exchange market. J. Intellect. Capital 8, 256–71. 10.1108/14691930710742835

[B65] Yi A. Davey H. Eggleton I. R. C. (2011). The effects of industry type, company size and performance on Chinese companies, IC disclosure: a research note. Australasian Account. Business Finance J. 5, 107–116.

[B66] Zeghal D. Maaloul A. (2010). Analysing value added as an indicator of intellectual capital and its consequences on company performance. J. Intellect. Capital 11, 39–60. 10.1108/14691931011013325

[B67] Zehri C. Abdelbaki A. Bouabddellah N. (2012). How intellectual capital affects firm's performance? Austral. J. Business Manage. Res. 2, 24–31.

